# Aerobic exercise training mitigates tumor growth and cancer-induced splenomegaly through modulation of non-platelet platelet factor 4 expression

**DOI:** 10.1038/s41598-023-47217-2

**Published:** 2023-12-11

**Authors:** Gabriel C. Tobias, João L. P. Gomes, Larissa G. Fernandes, Vanessa A. Voltarelli, Ney R. de Almeida, Paulo R. Jannig, Rodrigo W. Alves de Souza, Carlos E. Negrão, Edilamar M. Oliveira, Roger Chammas, Christiano R. R. Alves, Patricia C. Brum

**Affiliations:** 1 School of Physical Education and Sport, Universidade de São Paulo, Avenida Professor Mello Moraes, 65-Butantã, São Paulo, SP 05508-030 Brazil; 2 Children’s Cancer and Blood Foundation Laboratories, Departments of Pediatrics, and Cell and Developmental Biology, Drukier Institute for Children’s Health, Meyer Cancer Center, Weill Cornell Medicine, New York, NY USA; 3 Department of Surgery, Beth Israel Deaconess Medical Center, Harvard Medical School, Boston, MA 02115 USA; 4 Department of Physiology and Pharmacology, Karolinska Institutet, 171 77 Stockholm, Sweden; 5 Department of Radiology and Oncology, Faculdade de Medicine, Universidade de São Paulo, São Paulo, Brazil; 6 Department of Physiology & Biophysics, Institute of Biomedical Sciences, Universidade de São Paulo, São Paulo, Brazil; 7 Instituto do Coração (InCor), Hospital das Clínicas HCFMUSP, Faculdade de Medicina, Universidade de São Paulo, São Paulo, Brazil

**Keywords:** Cancer prevention, Prognostic markers

## Abstract

Exercise training reduces the incidence of several cancers, but the mechanisms underlying these effects are not fully understood. Exercise training can affect the spleen function, which controls the hematopoiesis and immune response. Analyzing different cancer models, we identified that 4T1, LLC, and CT26 tumor-bearing mice displayed enlarged spleen (splenomegaly), and exercise training reduced spleen mass toward control levels in two of these models (LLC and CT26). Exercise training also slowed tumor growth in melanoma B16F10, colon tumor 26 (CT26), and Lewis lung carcinoma (LLC) tumor-bearing mice, with minor effects in mammary carcinoma 4T1, MDA-MB-231, and MMTV-PyMT mice. In silico analyses using transcriptome profiles derived from these models revealed that platelet factor 4 (Pf4) is one of the main upregulated genes associated with splenomegaly during cancer progression. To understand whether exercise training would modulate the expression of these genes in the tumor and spleen, we investigated particularly the CT26 model, which displayed splenomegaly and had a clear response to the exercise training effects. RT-qPCR analysis confirmed that trained CT26 tumor-bearing mice had decreased Pf4 mRNA levels in both the tumor and spleen when compared to untrained CT26 tumor-bearing mice. Furthermore, exercise training specifically decreased Pf4 mRNA levels in the CT26 tumor cells. Aspirin treatment did not change tumor growth, splenomegaly, and tumor Pf4 mRNA levels, confirming that exercise decreased non-platelet Pf4 mRNA levels. Finally, tumor Pf4 mRNA levels are deregulated in The Cancer Genome Atlas Program (TCGA) samples and predict survival in multiple cancer types. This highlights the potential therapeutic value of exercise as a complementary approach to cancer treatment and underscores the importance of understanding the exercise-induced transcriptional changes in the spleen for the development of novel cancer therapies.

## Introduction

Cancer progression is significantly influenced by the complex interplay between tumor cells and the host, making the understanding of tumor-host crosstalk crucial for improving patient survival and well-being^1–3^. Exercise training is a non-pharmacological strategy known to improve health and well-being across diverse tissue systems, with numerous studies highlighting its beneficial effects on slowing down tumor growth in various murine cancer models^4–11^ and reducing the incidence of 13 different types of human cancers^12^. Exercise elicits beneficial adaptations in multiple tissues, impacting whole-body physiology through the secretion of diverse molecules, such as growth factors^13–15^, cytokines^13^, microRNAs^16,17^, and metabolites^18,19^.

The spleen, the largest lymphoid organ, plays a crucial role in modulating cancer risk by controlling hematopoiesis and immune responses^20,21^. Cancer-induced splenomegaly is associated with alterations in host hematopoiesis, characterized by leukocytosis, lymphopenia, and granulocytosis^22–24^. The tumor microenvironment influences splenomegaly by promoting the expansion of myeloid-derived suppressor cells (MDSCs) in the bone marrow, spleen, and lymph nodes through the production of granulocyte colony-stimulating factor (G-CSF)^22–26^.

Given the significant role of the spleen in cancer progression, understanding the transcriptional changes occurring within the organ is critical for developing effective therapeutic strategies. These transcriptional changes modulate cancer-induced splenomegaly, the immune response, and hematopoiesis, all of which have major implications for tumor growth and metastasis^3,27–29^. A comprehensive understanding of the molecular mechanisms underlying these transcriptional alterations is essential for elucidating the spleen's contribution to cancer progression and identifying potential therapeutic targets.

In this context, exercise has emerged as a potential modulator of the spleen's transcriptional profile, with studies demonstrating its profound impact on immune function^28,30^. Exercise not only increases the mobilization and redistribution of natural killer (NK) cells from the spleen to the tumor microenvironment^31^ enhancing the anti-tumor immune response, but also reduces cancer-induced splenomegaly by decreasing the production of pro-tumoral MDSCs in the spleen and increasing the mobilization of anti-tumoral immune cells from the spleen to the circulation^32^. This highlights the potential role of exercise in modulating the spleen's function during cancer progression.

In the current study, we aimed to uncover the transcriptional changes in the spleen induced by aerobic exercise training and their implications for tumor progression and splenomegaly in different murine cancer models. Our findings reveal a strong connection between exercise training and the modulation of key transcriptional events in the spleen, providing valuable insights into the molecular mechanisms through which exercise exerts its anti-tumor effects. This highlights the potential therapeutic value of exercise as a complementary approach to cancer treatment and underscores the importance of understanding the exercise-induced transcriptional changes in the spleen for the development of novel cancer therapies.

## Results

### Exercise training reduces tumor growth and attenuates cancer-induced splenomegaly.

**Figure 1:**
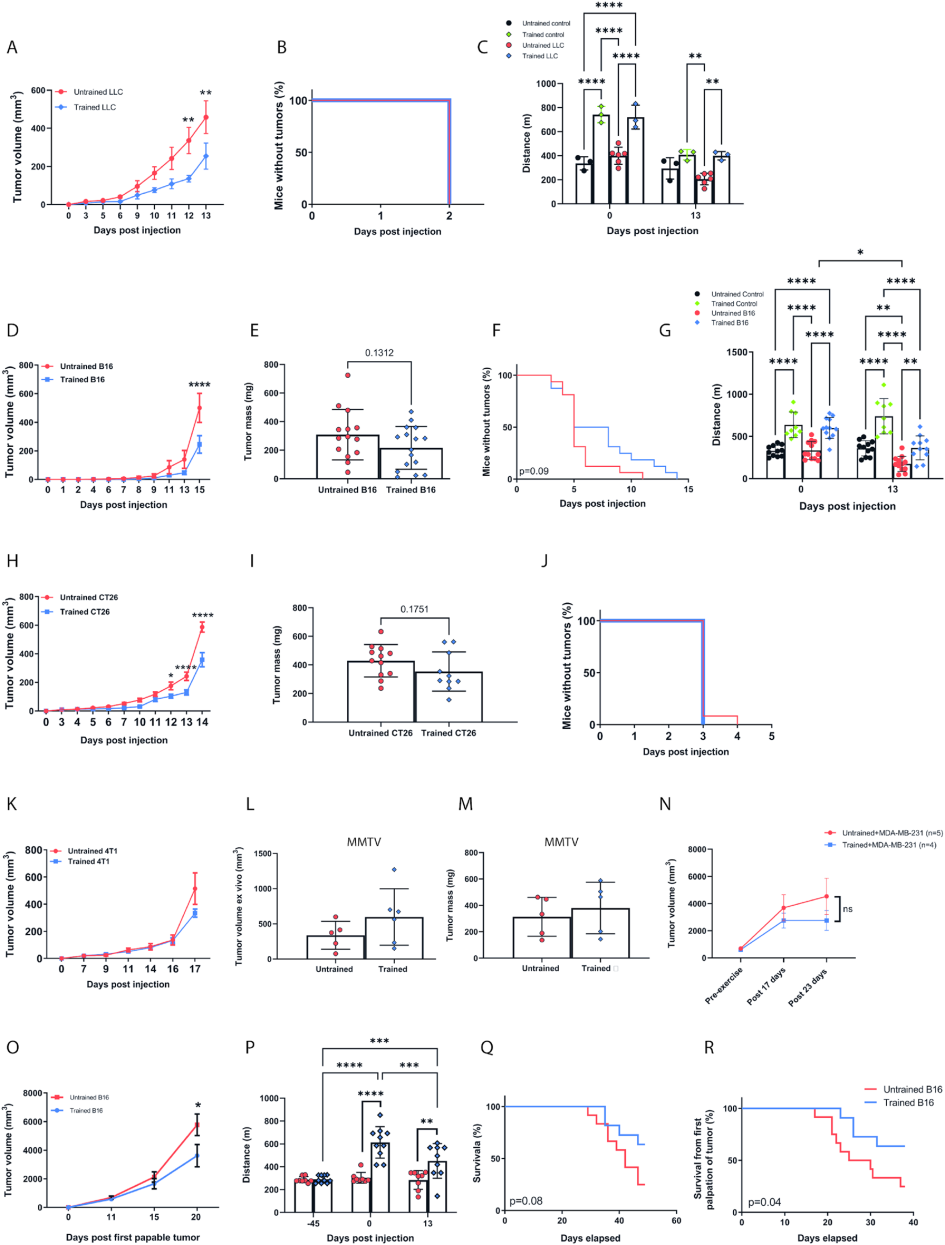
Exercise training reduces tumor growth and cancer-induced splenomegaly in multiple cancer models and attenuates cancer-induced exercise intolerance in tumor-bearing mice. (**A–C**) Tumor growth, percentage of mice without tumor, and distance until exhaustion during a maximal incremental running test in LLC tumor-bearing mice. (**D–G**) Tumor growth, tumor mass, and percentage of mice without tumor, and distance until exhaustion during a maximal incremental running test in B16F10 tumor-bearing mice. (**H–J)** Tumor growth, tumor mass, percentage of mice without tumor in CT26 tumor-bearing mice. (**K**) Tumor growth in 4T1 tumor-bearing mice. (**L,M**) Ex vivo tumor volume and tumor mass in MMTV-PyMT mice. (**N**) Tumor growth in mice orthotopically injected with MDA-MB-231 human breast cancer cell line. Data are presented as mean ± SD. n = 6–16. Repeated measures Two-Way ANOVA followed by post-hoc Tukey’s, or two-tailed Student’s t-test, or Two-Way ANOVA followed by post-hoc Tukey’s were used to compare differences between the variables. *p < 0.05, **p < 0.01, ***p < 0.001, ****p < 0.0001. Exercise training prolongs lifespan in B16F10 tumor-bearing mice. (**O–R**) Tumor growth from the first tumor palpation, distance until exhaustion during a maximal incremental running test, survival rate, and survival rate from the first tumor palpation in B16F10 tumor-bearing mice. B16F10 sedentary (n = 12); B16F10 trained (n = 11). Data were analyzed by Mantel–Cox log-rank test. The individual dots represent the data points from each mouse.

We first characterized protocols of aerobic exercise training in healthy C57BL/6 and BALB/c mice. Healthy C57BL/6 and Balb/c mice were submitted to a maximum incremental treadmill running test pre- and post-45 days of free access to a wheel running to determine whether a simple voluntary running protocol would be effective to increase aerobic exercise capacity. C57BL/6 mice ran more than 2 km/day and had 47% increased exercise performance after 45 days of voluntary wheel running (Supplementary Fig. S1A, B). However, we found a lower and more variable daily voluntary wheel running distance in BALB/c mice and no increase in aerobic exercise capacity (Supplementary Fig. S1C, D), so for BALB/c mice we opted to perform a 30-days treadmill exercise training which improved aerobic exercise capacity and gastrocnemius citrate synthase activity^33^. After confirming that these exercise training protocols can improve aerobic capacity in each specific mouse strain, LLC or B16F10 tumor cells were subcutaneously injected into untrained and trained C57BL6 mice, and CT26 or 4T1 tumor cells were subcutaneously injected into untrained and trained BALB/c mice. We evaluated tumor growth in these four different cancer models to determine the effects of exercise training on tumor progression. Exercise training slowed down tumor growth in LLC (Fig. 1A), B16F10 (Fig. 1D), and CT26 (Fig. 1H) tumor-bearing mice with minor effects in 4T1 tumor-bearing mice (Fig. 1K). We did not observe changes in time to the first palpable tumor identification (Fig. 1B, F, J, Supplementary Fig. S2C). We also evaluated tumor mass for the B16F10 (Fig. 1E), CT26 (Fig. 1I), and 4T1(Supplementary Fig. S2B) models, and although exercise reduced tumor volume in the B16F10 and CT26 models, tumor mass was not altered in both models. Since exercise was not able to reduce 4T1 tumor growth, we analyzed whether our result was not because we injected the tumor cells subcutaneously and not orthotopically. To evaluate whether exercise can reduce breast cancer tumor growth, we used MMTV-PyMT mice that develop breast cancer spontaneously, and we also used human breast cancer cells MD-MB-231 injected directly into the mammary fat pad of NUDE mice, however, no difference was observed in tumor growth and tumor mass as well as for tissue mass of different organs analyzed (Fig. 1L,M, and Supplementary Fig. S2E–M). Both untrained and trained tumor-bearing mice experience a decline in aerobic capacity due to the presence of the tumor, and the magnitude of loss is similar between the groups. However, the trained tumor-bearing mice retain a level of aerobic capacity comparable to that of untrained mice without cancer. (Fig. 1C, G, Supplementary Fig. S2D, and^33^). These findings are consistent with previous studies demonstrating that exercise training associated or not with pharmacological therapies can slow down the tumor progression, mainly when the exercise protocol started prior to the tumor cell injection^5,10,31^. We also set to evaluate the survival rate in untrained and trained B16F10 tumor-bearing mice in a 50-day follow-up. Again, exercise was able to reduce tumor growth and attenuate the loss of aerobic capacity in the mice (Fig. 1O, P). Only 4 out of 11 trained B16F10 tumor-bearing mice died throughout the 50-day follow-up, while 9 out of 12 untrained B16F10 tumor-bearing mice (75%) died in the same 50-day period. Log-rank test revealed that exercise training significantly prolonged lifespan from the first tumor palpation time point (Fig. 1Q, R). Since intrinsic aerobic capacity is suggested to be an important marker for the development and progression of different types of cancer as well as other diseases^12,34–39^, we evaluated whether intrinsic aerobic capacity could be associated with tumor growth. To exclude any effect of exercise on tumor growth, we evaluated only untrained mice and correlated the maximal distance achieved in the pre-test with tumor volume at the time of mice euthanasia. No association was found between the maximal distance achieved in the maximal test and tumor volume for all models used (Supplementary Fig. S3A–H). Furthermore, no association was also observed even when all animals were analyzed together (Supplementary Fig. S3I–J). Therefore, these data demonstrate that exercise training reduces tumor growth in different cancer models and prolongs survival in B16F10 tumor-bearing mice and that the tumor growth reduction is not associated with an intrinsic aerobic capacity of the mice. Together, these data indicate that exercise training is a powerful tool in counteracting tumor progression in multiple cancer models, and elucidating the mechanisms behind these benefits is highly scientifically relevant.

**Figure 2:**
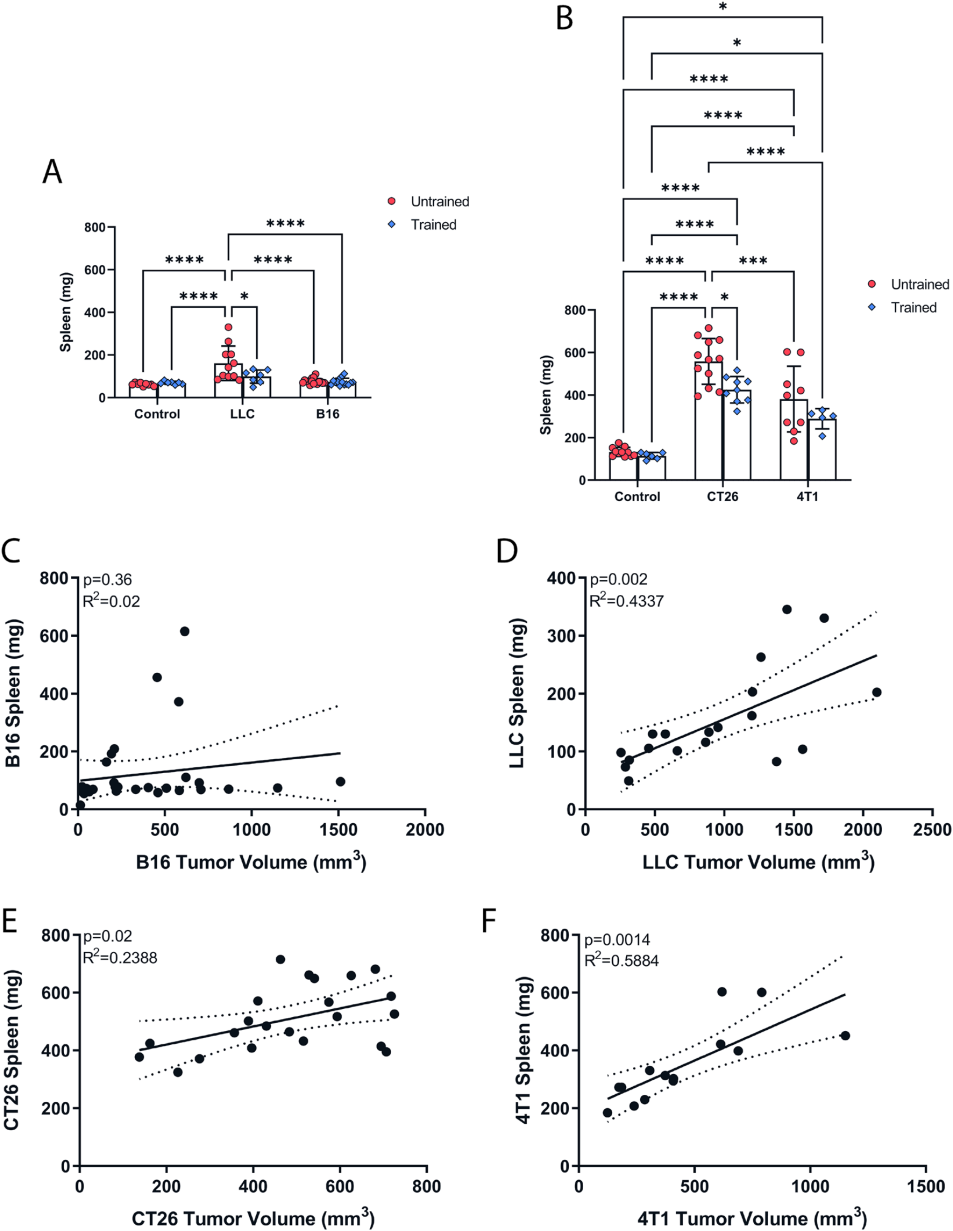
Exercise training reduces cancer-induced splenomegaly in multiple cancer models. Spleen mass in untrained and trained C57BL/6 (**A**) and Balb/c (**B**) mice. (**C–E**) Correlation analysis between spleen mass and tumor volume. Data are presented as mean ± SD. n = 6–16. One-Way ANOVA followed by post-hoc Tukey’s was used to compare differences between the variables. *p < 0.05, **p < 0.01, ***p < 0.001, ****p < 0.0001. The individual dots represent the data points from each mouse.

Cancer is a systemic disease that affects organs distant from the primary tumor. Similarly, the beneficial effects of exercise are related to acute and chronic adaptations of different organs to exercise. Given that both cancer and exercise affect different organs, we looked at the tissue mass of different organs to understand which tissues exercise might be protecting against the deleterious effects of cancer. Despite normal spleen mass in B16F10 tumor-bearing mice, we observed that all other cancer models induced splenomegaly (Fig. 2A, B). Exercise training attenuated cancer-induced splenomegaly in both LLC and CT26 models, whereas no significant changes were observed in the 4T1 model (Fig. 2A, B). For the other tissues analyzed, only the gastrocnemius and liver showed a significant reduction and increase, respectively, in their mass as a result of the CT26 and 4T1 tumors (Supplementary Fig. S4A–4W), however, exercise was not able to attenuate this reduction and increase (Supplementary Fig. S4S, W). The CT26 model showed a significant reduction in gastrocnemius and tibialis anterior mass, and despite exercise training intervention, the loss was not reversed^33^. Interestingly, we observed a positive correlation between spleen mass and tumor volume in LLC, CT26, and 4T1 tumors, whereas no correlation was observed in the B16F10 model. In summary, these findings indicate that exercise training can reduce cancer-induced splenomegaly (Fig. 2C–F).

### Identification of differential transcriptional patterns in spleen and tumor of tumor-splenomegaly mice

**Figure 3:**
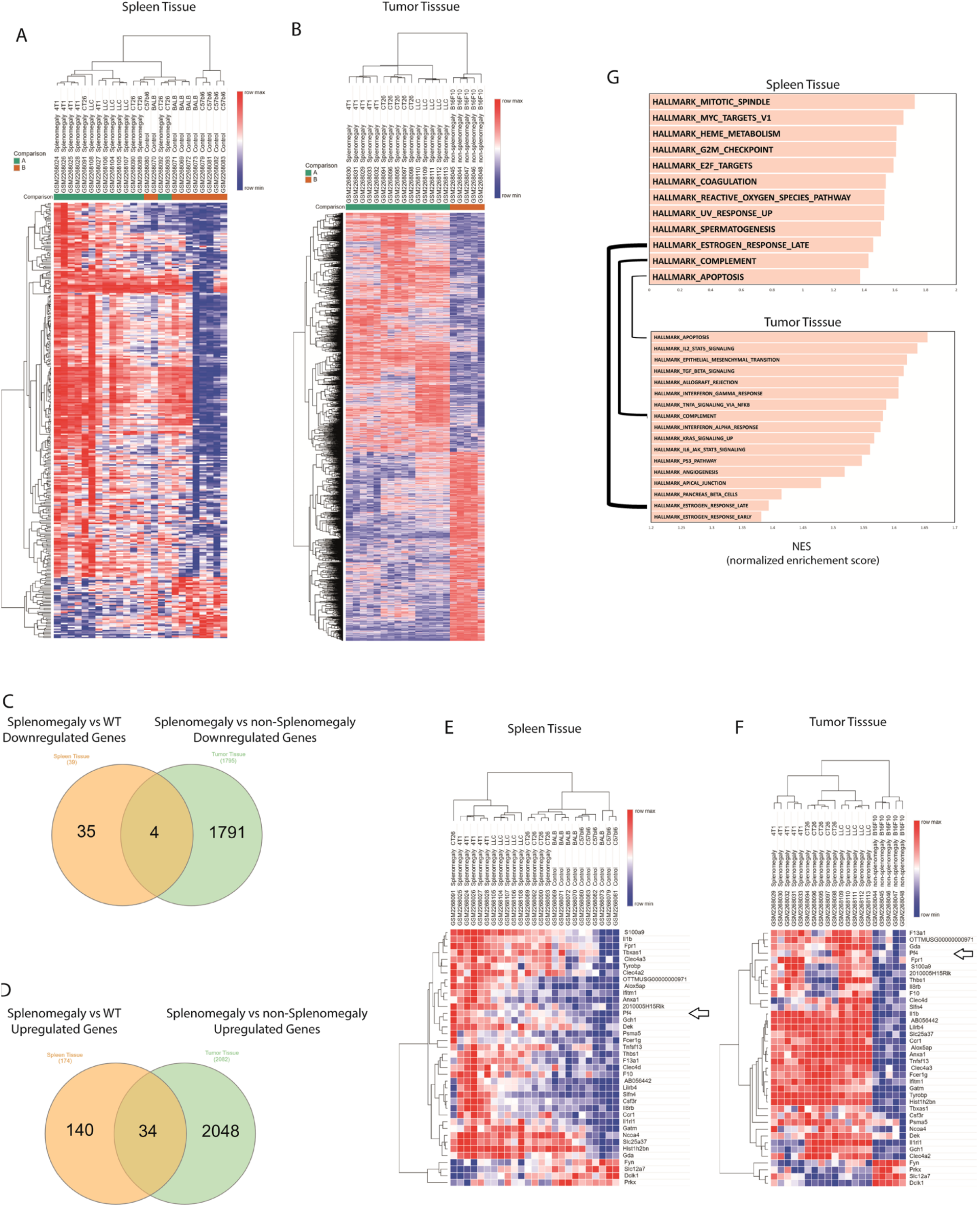
Transcriptional changes in the spleen and tumor of tumor-splenomegaly mice. (**A,B**) Representative heat maps are showing the hierarchical clustering analysis of spleen (splenomegaly vs control) and tumor (splenomegaly vs non-splenomegaly) messenger RNA expression of tumor-bearing mice. Significance was defined by false discovery rate (FDR) q < 0.05. (**C,D**) Venn diagrams of downregulated and upregulated genes shared among spleen and tumor from tumor-splenomegaly mice. (**E,F**) Representative heat maps are showing the upregulated genes shared among spleen and tumor from tumor-splenomegaly mice. (**G**) The gene sets enriched and shared among spleen and tumor from tumor-splenomegaly mice (4T1, CT26 and LLC) determined by GSEA using Hallmark gene set. Significance was defined by false discovery rate (FDR) q < 0.25.

Spleen plays an important role in the immune response^3,40,41^ and antitumor immune response^2,3^. Moreover, the mechanisms responsible for cancer-induced splenomegaly are not fully understood. To explore the transcriptional changes that drive this phenotype, we compared the spleen transcriptome from cancer models with splenomegaly (LLC, CT26, and 4T1) with control mice using the GSE85507 dataset from the NCBI Gene Expression Omnibus (GEO) public repository. We observed 39 downregulated and 174 upregulated genes across all these cancer models compared to controls (Fig. 3A and Supplementary Table S1). Taking into consideration that tumor progression may play a primary role to induce splenomegaly, we took advantage of the same dataset described above (GSE85507) to compare the transcriptional signature of the tumors from cancer models with splenomegaly (4T1, CT26, and LLC tumors) with tumors from the B16F10 model that did not display splenomegaly. We observed 1795 downregulated genes and 2082 upregulated genes in tumors from mice with splenomegaly (Fig. 3B and Supplementary Table S2). Finally, we found 4 downregulated (Fig. 3C–E) and 34 upregulated (Fig. 3D–F) genes shared by both the spleen and tumor transcriptomes from the analysis described above. We next applied GSEA using the Hallmark database to assess potentially altered biological pathways in the spleen and tumor. Notably, pathways enriched in both spleen and tumor analysis were only those related to estrogen response late, apoptosis, and complement (Fig. 3G).

**Figure 4:**
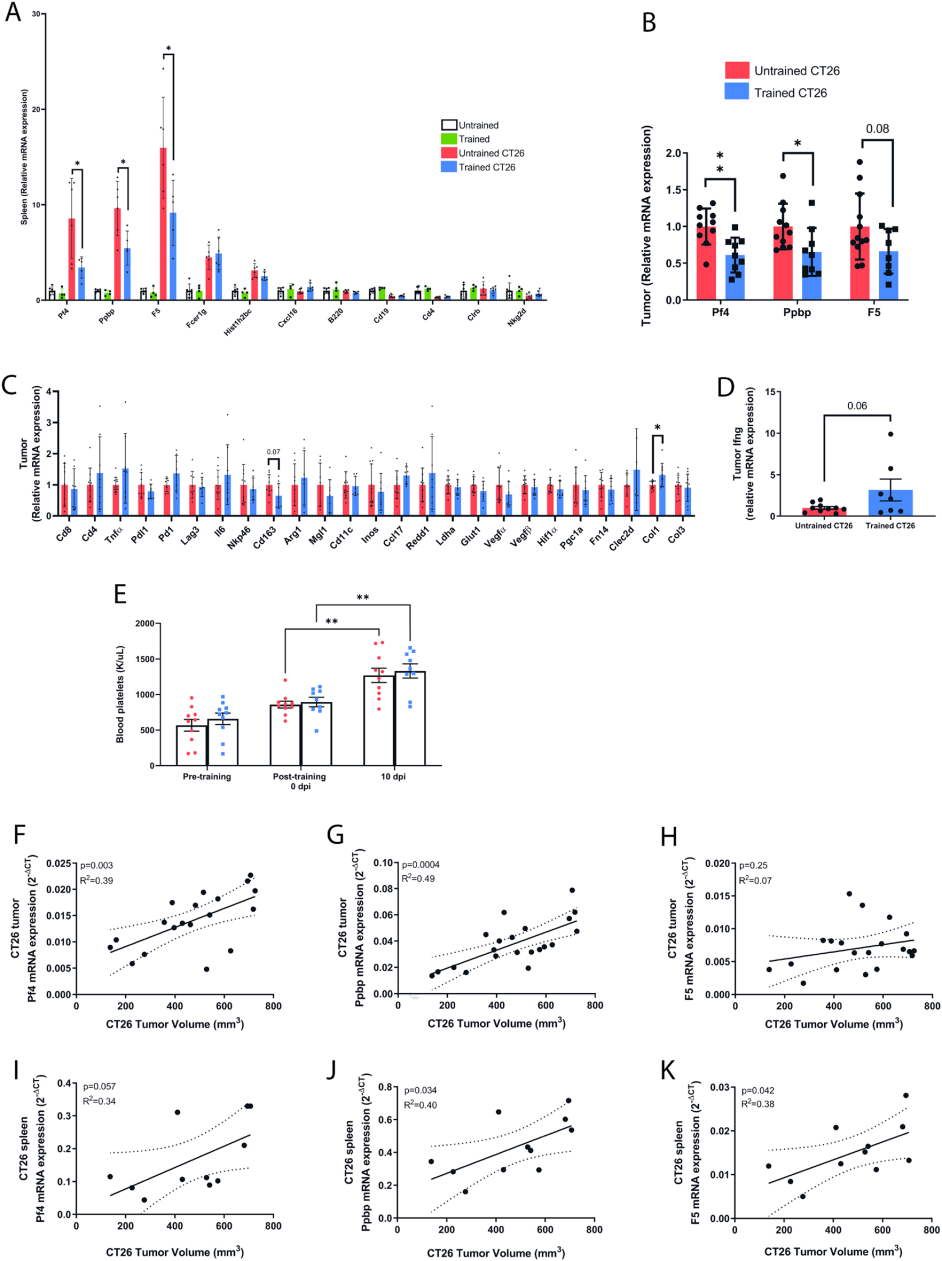
Exercise training reduces the Pf4, Ppbp, and F5 mRNA levels in both the spleen and tumor of CT26 tumor-bearing mice. (**A**) mRNA expression in the spleen of untrained and trained CT26 tumor-bearing mice. (**B–D**) mRNA expression in the tumor of untrained and trained CT26 tumor-bearing mice. **E**: Blood platelet count was analyzed in baseline (before exercise training—non-tumor), post-training (30 days of exercise training—non-tumor), and post-10 days of CT26 tumor cell injection (CT26 tumor-bearing mice). (**F–H**) Correlation analysis between Pf4, Ppbp, and F5 mRNA expression in the tumor and tumor volume. (**I–K**) Correlation analysis between Pf4, Ppbp, and F5 mRNA expression in the spleen and tumor volume (**D**). n = 4–12. Data are presented as mean ± SD. Repeated measures Two-Way ANOVA followed by post-hoc Tukey’s, or two-tailed Student’s t-test were used to compare differences between the variables. *p < 0.05, **p < 0.01, ***p < 0.001, ****p < 0.0001. The individual dots represent the data points from each mouse.

Considering that Pf4 stimulates CT26 tumor growth in vivo^42^, has a critical role in the complement processes pathway, and exhibits altered protein expression in plasma due to exercise^43^, and given that CT26 represents the cancer model with the most significant splenomegaly and well-defined responses to our exercise training protocols, we investigated whether Pf4 mRNA expression might be correlated with tumor growth and whether exercise training could influence Pf4 expression in the spleen and tumor. RT-qPCR analysis revealed that Pf4 mRNA expression was increased by more than fivefold in the spleen of CT26 tumor-bearing mice as compared to healthy control mice (Fig. 4A), and exercise training significantly reduced the Pf4 mRNA levels towards the control levels (Fig. 4A). As Pro-platelet basic protein (Ppbp) and Coagulation factor V (F5) are Pf4 partners with direct protein–protein interactions, and both are upregulated in the spleen of splenomegaly mice (Supplementary Table S1), we analyzed mRNA expression of both. Similar to Pf4, Ppbp, and F5 mRNA expression was increased by more than fivefold in the spleen of CT26 tumor-bearing mice as compared to healthy control mice (Fig. 4A) and exercise training significantly reduced the Ppbp and F5 mRNA levels towards the control levels. Fcer1g, another gene shared by both the spleen and tumor transcriptomes (Fig. 3D-3F), was upregulated more than fourfold in the spleen of CT26 tumor-bearing mice as compared to healthy control mice (Fig. 4A), however, exercise training did not change Fc receptor, IgE, high affinity I, gamma polypeptide (Fcer1g) mRNA levels (Fig. 4A). Consistent with transcriptional changes in the spleen, exercise training also decreased Pf4 and Ppbp*,* and F5 mRNA levels in the tumor tissue (Fig. 4B). Additional RT-qPCR analysis revealed that the transcripts abundance of other immune cell types, endothelial cells, and metabolism remained unchanged in the spleen (Fig. 4A) and tumors of trained mice as compared to their untrained counterparts (Fig. 4C, D). Because Pf4 is known to be released during platelet activation, we analyzed blood platelets count at (1) baseline, (2) after the exercise training protocol and before tumor cell injection, and (3) after 10 days of CT26 cells injection. CT26 tumor-bearing mice had increased platelet counts, however, it was unaffected by exercise interventions (Fig. 4E). Finally, both tumor and spleen Pf4, Ppbp, and F5 mRNA expression were correlated with the CT26 tumor volume (Fig. 4F–K**)**. Importantly, these correlations are not found for spleen mass, suggesting that Pf4 expression levels in both spleen and tumor are associated only with tumor growth and not splenomegaly (Supplementary Fig. S5A–F). Altogether, these findings demonstrate that exercise training reduces the Pf4, Ppbp, and F5 mRNA levels in both the spleen and tumor, and this response is associated with decreased tumor growth in CT26 tumor-bearing mice.

### Pf4 predicts survival in cancer patients.

**Figure 5:**
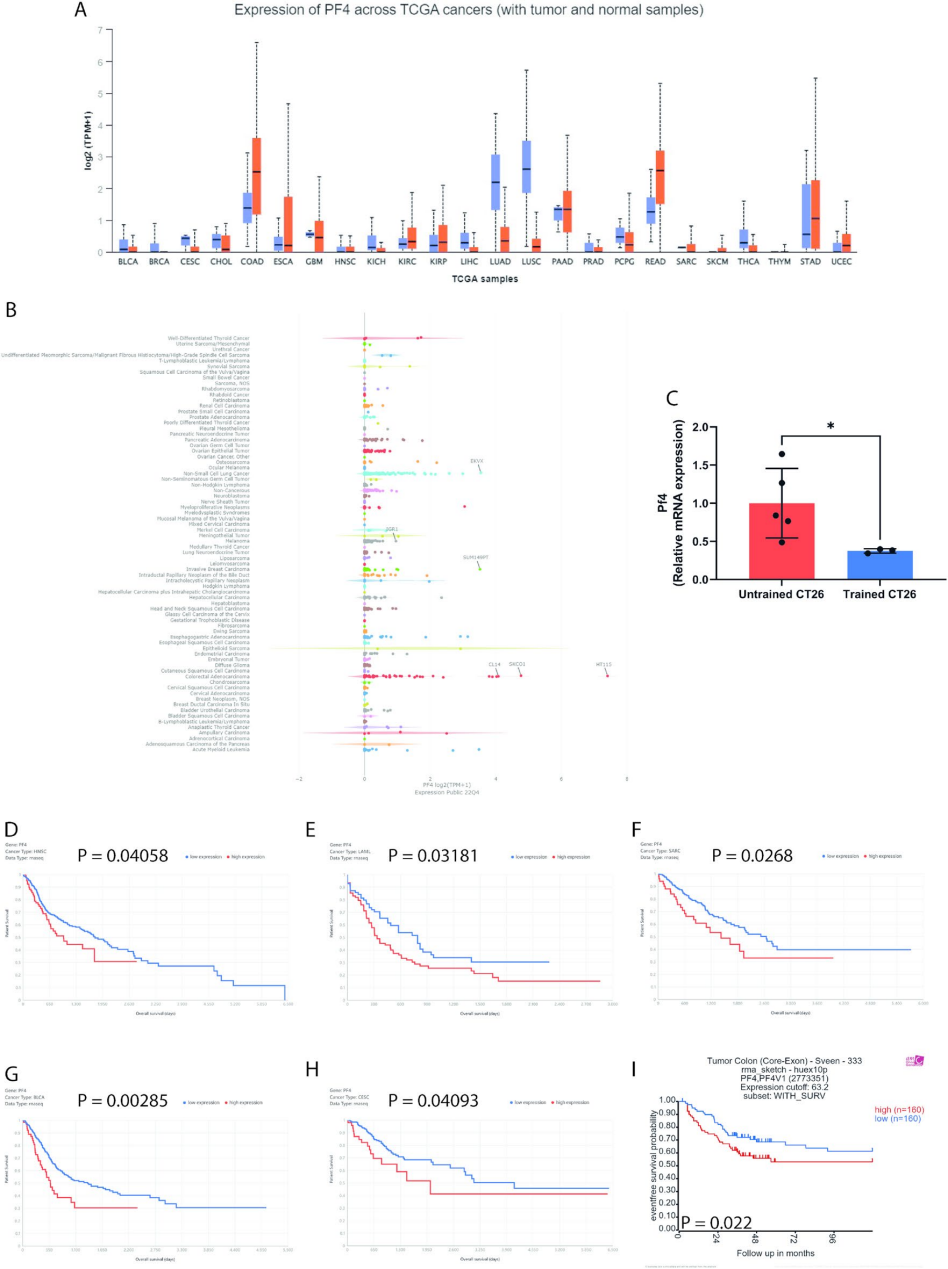
Exercise training reduces non-platelets Pf4 mRNA expression in CT26 primary tumor cells, and Pf4 predicts survival across TCGA cancers. (**A**) PF4 mRNA expression across TCGA cancers, calculated with ULCAN (http://ualcan.path.uab.edu/analysis.html). (**B**) PF4 mRNA expression across multiple human cancer cell lines, calculated with DepMap Portal (DepMap: The Cancer Dependency Map Project at Broad Institute). (**C**) Pf4 mRNA expression on CT26 primary tumor cells of untrained and trained tumor-bearing mice. Data are presented as mean ± SD. Two-tailed Student’s t-test was used to compare differences between the variables. *p < 0.05. (**D–H**) Overall survival across TCGA cancers with low PF4 (blue) and high PF4 (red) mRNA levels. Kaplan–Meier curves are generated by dividing patients into two groups and comparing the survival times between each group. The division was made based on the mean expression of the Pf4. *HNSC* head-neck squamous cell carcinoma, *LAML* acute myeloid leukemia; *SARC* sarcoma, *BLCA* bladder urothelial carcinoma, *CESC* cervical squamous cell carcinoma and endocervical adenocarcinoma. Kaplan–Meier analysis was performed on genetic determinants of cancer patient survival (tcga-survival.com). (**I**) Event-free survival of colon cancer patients with low PF4 (blue) and high PF4 (red) mRNA levels calculated with the ‘‘R2: tumor colon (core-exon)-Sveen-333-rna_sketch-huex10p’’ dataset (http://r2.amc.nl). Kaplan–Meier analysis was performed in R2: Genomics Analysis and Visualization Platform (http://r2.amc.nl). The individual dots represent the data points from each mouse.

Pf4 is downregulated in different types of cancer compared to healthy tissue in TCGA samples (Fig. 5A). In humans, Pf4 is almost exclusively expressed in the whole blood and to a lesser extent in the spleen and the lung (Supplementary Fig. S7A). Interestingly, using the BioGPS Gene Expression Atlas (biogps.org), we found that Pf4 is highly expressed in macrophages, bone, and bone marrow in mice (Supplementary Fig. S7B). More importantly, Pf4 Log2(TPM + 1) expression in human colon, breast, and lung cancer tumor cells (Fig. 5B) is similar to or even higher than Pf4 expression in tumor tissue of TCGA samples (Fig. 5A). To verify Pf4 expression in CT26 tumor cells, we compared Pf4 gene expression in CT26 tumors, in CT26 primary tumor cells, and in CT26 cells that were never injected into mice. Interestingly, CT26 cells that were never injected into mice do not express Pf4, whereas CT26 primary tumor cells express Pf4 more than 50 × compared to the CT26 cells (Supplementary Fig. S6A), showing that the tumor microenvironment induces increased Pf4 gene expression in CT26 tumor cells. Of note, Pf4 expression in CT26 tumors is more than 4000× higher than in CT26 primary tumor cells. (Supplementary Fig. S6A). Given that aspirin (ASP) treatment can inhibit Pf4 expression and release by platelets^44^, we examined whether platelet Pf4 exerts any influence on tumor growth and splenomegaly induced by the CT26 model. We treated mice 3 times a week for 4 weeks before the tumor cell injection and continued the treatment for another 12 days after the tumor cell injection. ASP treatment was not able to reduce tumor growth, splenomegaly, and increased liver mass induced by the CT26 tumor (Supplementary Fig. S6B–F). Of note, ASP treatment did not affect Pf4 expression in the CT26 tumor (Supplementary Fig. S6G) even though this reduced plasma Pf4 levels (Supplementary Fig. S6H), confirming that platelet Pf4 is not responsible for the increased Pf4 gene expression in the tumor microenvironment, and suggesting that the effects of exercise on Pf4 expression must be occurring in tumor cells, or in tumor-associated macrophages than in intratumoral platelets. Since the CT26 tumor is composed of only ~ 10% tumor-associated macrophages^45^, we analyzed whether exercise would reduce Pf4 expression directly in CT26 tumor cells. We generated a primary cell culture of CT26 tumors from untrained and trained mice. Interestingly, exercise was able to reduce Pf4 mRNA expression specifically in tumor cells (Fig. 5C). Together, these results reinforce that the effects of exercise on tumor Pf4 expression are occurring in part in tumor cells. To examine the clinical relevance of tumoral Pf4 expression, we next assessed the effect of Pf4 on multiple cancer patient survival. Impressively, we found that Pf4 is associated with survival in patients with different types of cancer in multiple cohorts analyzed (Fig. 5D–I and Supplementary Fig. S7C–Q).

## Discussion

Identifying new therapies to reduce cancer progression is a major research challenge. In the current study, we have demonstrated that exercise training reduces tumor growth in the B16F10, LLC, and CT26 cancer murine models. Although no difference was observed for the 4T1 model, this result may be a reflection of the low number of samples used, since many studies have shown that the exercised 4T1 tumor-bearing mice exhibited a significant decrease in tumor growth and splenomegaly^46^. Based on the existing literature, the exercise protocols were chosen to reflect clinically relevant exercise prescriptions while accounting for the physiological and biomechanical differences between humans and mice^47–49^. Studies have shown varying impacts of exercise intensity and duration on cancer progression, which guided our protocol design to elucidate the potential therapeutic and preventive effects of exercise training on tumor growth and cancer-induced splenomegaly across different mouse strains and cancer models^47–49^.These findings are consistent with previous studies demonstrating that exercise training associated or not with pharmacological therapies can slow down the tumor progression, mainly when the exercise protocol started prior to the tumor cell injection^5,10,31^. In addition, previous studies have shown that exercise training improves survival in different cancer models, including Hepa 1–6^50^ and C26^51^. Here, we found that exercise training also prolongs the lifespan in B16F10 tumor-bearing mice. Recently, Pedersen et al. (2016) demonstrated that exercise reduced B16F10 melanoma tumor growth in young and old mice and lung metastatic nodule formation^31^. Exercise performed in voluntary wheel running reduced tumor growth when performed only prior to tumor cell injection, and when performed prior to and maintained after tumor cell injection. Similarly, Buss et al. (2020) observed no change in B16F10 tumor growth when exercise was performed in a voluntary wheel running only after tumor cell injection^52^.

Recent studies have shown the beneficial effects of exercise on cancer-induced splenomegaly^32,46^. Here, we have observed that CT26, LLC and 4T1 tumor-bearing mice developed splenomegaly, and that exercise training was efficient in reducing it. Corroborating our findings, Pin et al. (2015) have demonstrated that six weeks of exercise training prior-tumor cell injection and 2 weeks of exercise training post-tumor cell injection reduced splenomegaly induced by the C26 tumor^53^. In addition, Mehl et al. (2005) also demonstrated that exercise training reduced splenomegaly in *Apc*^Min/+^ transgenic mice^54^. Crucially, exercise decreases cancer-induced splenomegaly effectively, exhibiting results comparable to those achieved through treatment with lurbinectedin (PM01183) and activin receptor type 2 (ACVR2) ligand inhibitors^27,55^.

The absence of liver metastasis promotion by CT26 and 4T1 subcutaneous tumors (data not shown) suggests that the observed increase in liver mass among CT26 and 4T1 mice may be associated with heightened hepatic fibrosis levels. This correlation is further supported by alterations in hepatic metabolic markers^56^, an increase in liver parenchyma^57^, and the presence of steatosis^58^.

Our study is the first to show the transcriptional chances in the spleen induced by tumor-promoting splenomegaly. Moreover, our study is the first to show the transcriptional differences in the tumor between tumor-splenomegaly mice (4T1, CT26, and LLC tumors) and tumor non-splenomegaly mice (B16F10). Most importantly, exercise training was also able to reduce the mRNA levels of Pf4 in the tumor and spleen, and this reduction was associated with attenuated tumor growth. Pf4 is described as a factor associated with platelets^59^. Elevated Pf4 levels in platelets or serum after platelet activation have been correlated with the occurrence of colorectal cancer^60^, pancreatic cancer^61^, breast adenocarcinoma, and osteosarcoma^62^. In tumors, Pf4-induced platelet accumulation can promote the acceleration of tumor growth, since platelets can modulate tumor cells and tumor microenvironment. Pf4 was identified as a circulating factor that correlates with patients’ survival when it is overexpressed in lung tumors^1^ and demonstrated to stimulate CT26 tumor growth in vivo^42^. Although Pf4 induces increased megakaryopoiesis restricted to the bone marrow^1^, Pf4 might induce increased spleen megakaryopoiesis in mice with other cancer types. In fact, when Pf4 is overexpressed in the liver, CT26 tumor-bearing mice displayed an additional CT26 tumor growth^42^. Together, these results suggest that Pf4 is a circulation factor that favors tumor growth.

In our study, we also examined the effects of aspirin (ASP) treatment on tumor growth and splenomegaly induced by the CT26 model. The results showed that ASP treatment did not reduce tumor growth, splenomegaly, and increased liver mass induced by the CT26 tumor. This result aligns with Zelenay's study, which demonstrated that ASP treatment alone does not decrease CT26 tumor growth^63^ but can reduce tumor growth when combined with anti-PD-1 therapy. Moreover, ASP treatment did not affect Pf4 expression in the CT26 tumor, even though it reduced plasma Pf4 levels. This finding suggests that platelet Pf4 is not responsible for the increased Pf4 gene expression in the tumor microenvironment, and indicates that the effects of exercise on Pf4 expression must be occurring in tumor cells or tumor-associated macrophages rather than intratumoral platelets.

Additionally, the available information on PF4 expression in tumor cells is still limited and may vary depending on the specific type of cancer. For example, in oligometastatic melanoma cells, Pf4 knockout increases cell migration in vitro and lung metastasis formation in vivo^64^. Similarly, in human head and neck cancer tumor cells, Pf4 overexpression reduces tumor growth and increases survival^65^. Interestingly, Pf4 knockdown in HCT116 and HT29 colon cancer cells reduces clonogenic growth and cell proliferation^66^. Although platelets Pf4 is well-studied, the mechanisms driving its production in platelets and in the tumor cells are unclear. For example, in plasmacytoid dendritic cells, the production of Pf4 is increased upon co-exposure to hypoxia and Tlr9 agonist both at the protein and the mRNA level via mtROS and HIF-2a^67^. Most importantly, in the areas of tumor hypoxia, elevated levels of Tlr9 are also observed^68–70^.

The effects of exercise on Pf4 expression are not well understood, as there has been limited research in this area. Moreover, the studies have been focused on understanding the effects of exercise on platelet-derived Pf4 in the bloodstream^43,71–77^. Interestingly, it has been shown that exercise training can improve tumor angiogenesis and that it can be associated with the presence of functional blood vessels irrigating the tumor, therefore decreasing the hypoxia-reperfusion cycle^10,78,79^. Furthermore, we found that exercise training significantly reduced the percentage of hypoxic areas within the CT26 tumor compared to the CT26 tumor of untrained mice as measured using Hypoxyprobe (data not shown). Therefore, these results suggest that exercise-induced reduction of tumor hypoxia may be one of the mechanisms involved in the reduction of Pf4 expression in CT26 tumor cells.

Finally, our analysis of the clinical relevance of tumoral Pf4 expression revealed that Pf4 is associated with survival in patients with various types of cancer. This finding highlights the potential of Pf4 as a prognostic marker and therapeutic target, corroborating previous suggestions by other studies^80,81^. The relationship between Pf4 expression and patient survival also underscores the potential benefits of exercise as an adjuvant therapy in cancer treatment.

In summary, our study provides valuable insights into the potential therapeutic effects of aerobic exercise training in cancer treatment through the modulation of non-platelet Pf4 expression. The results highlight the importance of further research to validate these findings in human cancer patients and to investigate the optimal duration, intensity, and type of exercise that would provide the most significant benefits for cancer patients. Moreover, future research should aim to elucidate the precise molecular pathways involved in the exercise-induced modulation of Pf4 expression and explore the potential of exercise training as an adjuvant therapy for cancer patients, taking into account individual patient characteristics and cancer types.

## Conclusion

In conclusion, our study demonstrates that exercise training effectively improves aerobic capacity in specific mouse strains and reduces tumor growth in various cancer models. Importantly, exercise training also prolongs survival in B16F10 tumor-bearing mice, regardless of the mice's intrinsic aerobic capacity. Moreover, exercise training alleviated cancer-induced splenomegaly in multiple cancer models. The transcriptional changes driving cancer-induced splenomegaly were investigated, revealing numerous differentially expressed genes in both spleen and tumor tissue. Importantly, exercise training was found to reduce Pf4, Ppbp, and F5 mRNA levels in both the spleen and tumor, which was associated with decreased tumor growth in CT26 tumor-bearing mice. Pf4 expression was also found to be upregulated in various types of human cancer compared to healthy tissue. The results indicate that the tumor microenvironment can induce increased Pf4 gene expression, with exercise training capable of reducing Pf4 expression in tumor cells. Furthermore, a strong association was observed between Pf4 expression and patient survival across different cancer types. Altogether, these findings emphasize the potential therapeutic value of exercise in modulating tumor progression and underscore the importance of understanding the underlying molecular mechanisms to develop effective treatment strategies.

### Limitation

The subcutaneous injection of tumor cells derived from non-subcutaneous tumors, such as LLC or CT26, should indeed be acknowledged as a limitation of the model. This is due to the vastly different immune microenvironments present in the skin compared to the host organs from which these tumor types originate^82–84^. For instance, subcutaneous models do not fully replicate the tumor's original microenvironment, leading to a potential misrepresentation of the tumor's interaction with its surroundings and the host immune response^82–84^. Moreover, subcutaneous models are known to be less aggressive and less metastatic compared to orthotopic models, which could undermine the understanding of tumor progression and the assessment of potential therapeutic approaches^82–84^. The divergent immune responses could significantly affect the outcomes, particularly when studying immunotherapies or the impact of exercise training on tumor growth, as it might not accurately reflect the scenario in the host organs.

In addition, cancer predominantly affects older individuals, and the exercise response may vary with age. While our study focused on adult mice, several studies have demonstrated similar exercise-induced benefits in older mice^85^.

## Methods

### Ethics and cancer models

This study was approved by the Ethical Committee of School of Physical Education and Sport, University of Sao Paulo (protocols #2014/08, #2015/03 and #115/16). All animal procedures were performed in accordance with the Guide for the Care and Use of Laboratory Animals (National Institutes of Health, USA), and with ethical principles in animal research adopted by the Brazilian Council for the Control of Animal Experimentation. This study is reported in accordance with ARRIVE guidelines.

Eight to twelve-week-old male C57BL/6, and female Balb/c and female NUDE mice were used in this study. Female MMTV (Mouse Mammary Tumor Virus Polyoma Middle T antigen; MMTV-PyMT) mice that represent transgenic mice with spontaneous breast tumor appearance were also studied. Tumors spontaneously appeared in the MMTV-PyMT mice between week 10 and week 13 of age^86^. The Medical School of the University of Sao Paulo animal facility provided the mice. Mice were housed in an animal facility under controlled temperature (22 °C) with 12:12 h light:dark cycle and had ad libitum assessment to standard laboratory chow and water. Tumor cells from melanoma B16F10, colon tumor 26 (CT26), Lewis lung carcinoma (LLC), and mammary carcinoma 4T1 were used in this study. B16F10, LLC and MDA-MB-231 cells were cultured in Dulbecco's modified Eagle's medium (DMEM; Gibco, NY, US) medium supplemented with 10% fetal bovine serum (Gibco, NY, US) and 1% penicillin–streptomycin at 37 °C with 5% CO_2_. CT26 and 4T1 cells were cultured in RPMI-1640 (Gibco, NY, US) medium supplemented with 10% fetal bovine serum (Gibco, NY, US) and 1% penicillin–streptomycin at 37 °C with 5% CO_2_. Mice were injected subcutaneously in the right flank with 10^6^ tumor cells diluted in 100 µL of PBS. C57BL6 mice were injected with B16F10 or LLC tumor cells, while Balb/c mice were injected with CT26 or 4T1 tumor cells. B16F10, CT26, LLC, and 4T1 cells were kindly provided by Dr. Roger Chammas (Department of Radiology and Oncology, School of Medicine, University of São Paulo, São Paulo, Brazil). Tumor volume was measured daily after tumor cell injections using a caliper. The largest and smallest diameter of the tumor were measured as described by Bergers et al. (2003)^87^. Values obtained were used in the following formula for estimating tumor volume: 1/2(length × width^2^)^87,88^. For ethical purposes and endpoint, mice were sacrificed when tumor volume reached approximately 500–600 mm3. Mice with necrotic tumors were euthanized and excluded from further analysis. Mice were sacrificed by cervical dislocation under isoflurane anesthesia. Tissues were carefully harvested and weighed and cut into two pieces and stored at − 80 °C for further RNA extraction.

### CT26 primary tumor cells

CT26 tumors were mechanically disaggregated into small pieces of approximately 1 mm^3^ in a culture plate. After disaggregation, the tumors were maintained in RPMI culture medium supplemented with 10% fetal bovine serum and 1% penicillin/streptomycin in a 5% CO incubator_2_ at 37 °C. The cells were continuously maintained in culture for 7 days in the presence of 1.0 μg/mL puromycin for the selection of a population of CT26 tumor cells, as this exhibits resistance cassette for puromycin. After 7 days, primary CT26 tumor cells were maintained in RPMI culture medium supplemented with 10% fetal bovine serum and 1% penicillin/streptomycin in a 5% CO incubator_2_ at 37 °C.

### Survival

C57BL6 mice performed exercise training in voluntary running wheels for 95 days (45 days before B16F10 tumor cells injection + 50 days after B16F10 tumor cells injection). Each mouse was housed in individual cages with free access to a running wheel for small animals (Silent Spinner, Kaytee) with a magnetic sensor to determine the distance each mouse traveled daily during the experiment.

Mice were palpated twice daily for evidence of a tumor. If the presence of a tumor was confirmed, mice were examined daily. For ethical purposes and endpoint, mice were sacrificed when they appeared moribund, indicating a low probability of surviving for more than 24 h.

### Maximal incremental running test

Mice were submitted to incremental exercise testing on a motorized treadmill. A running test was performed initially to assess baseline performance, after exercise training protocol, and after tumor cell injection (figures show the specific day of the running test for each model). Mice were acclimated to the treadmill for four consecutive days prior to the treadmill performance test. Acclimation consisted of 5 min of running at 6 m/min followed by 5 min at 6–12 m/min. For performance evaluation, mice warmed up for 3 min at 6 m/min. After this point, speed was increased by 3 m/min every 3 min at 0% grade until exhaustion was reached. The maximal speed achieved by each mouse and the time to exhaustion were recorded. Mice were encouraged to continue running by gentle taps and classified as exhausted when they could no longer keep running despite prodding. This test provided the total distance run, and the peak workload was measured at the termination of the test. Based on the latter, individual workloads corresponding to 60% peak workload were determined^89^.

### Exercise training protocols

C57BL6 mice performed exercise training in voluntary running wheels for 60 days (45 days before tumor cells injection + 15 days after tumor cells injection). Each mouse was housed in individual cages with free access to a running wheel for small animals (Silent Spinner, Kaytee) with a magnetic sensor to determine the distance each mouse traveled daily during the experiment. Because wheel running did not increase aerobic exercise capacity in Balb/c, Balb/c mice were exercised using a treadmill training protocol. Exercise training consisted of 6 weeks of running (4 weeks before tumor cell injection + 15 days after tumor cell injection) on a motor treadmill (AVS, Campinas, Brazil), 5 days/week, for 60 min at 60% of the maximal intensity achieved in a maximal incremental running test as previously described^89^. The MMTV-PyMT mice performed exercise training on a motor treadmill (AVS, Campinas, Brazil) for 8 weeks, 5 days/week, for 60 min at 60% of the maximal intensity achieved in a maximal incremental running test as previously described^89^. Nude mice performed exercise training on a motorized treadmill for 21 days after MDA-MB-231 tumors achieved ~ 500 mm^3^.

### Quantitative real-time PCR

RNA was isolated from frozen tumor and spleen samples using *RNeasy Mini Kit* (Qiagen) and reverse transcribed using high-capacity cDNA Reverse Transcription kit (Thermo Fisher Scientific, USA) accordingly to the manufacturer protocols. Before cDNA synthesis, RNA purity and concentration were determined spectrophotometrically by NanoDrop 2000 (Thermo Fisher Scientific, USA) and RNA integrity was checked by 1% agarose gel stained with Nancy-520 (Sigma-Aldrich, USA). cDNA was analyzed by quantitative real time PCR (RT-qPCR). Reactions were performed using 10 ng of cDNA and 300 nM of each primer mixed with SYBR Green PCR Master Mix (Thermo Fisher Scientific, USA). Results were expressed using the comparative cycle threshold (Ct) method as described by the manufacturer. The ΔCt values were calculated in every sample for each gene of interest as Ct gene of interest minus Ct housekeeping, using Cyclophilin A and TATA box binding protein (Tbp) as housekeeping, and expressed repetitive elements Orr1a0 to spleen samples. The calculation of the relative changes in the expression level of one specific gene (ΔΔCt) was performed by subtraction of the average ΔCt from the Control group to the ΔCt from each sample, and fold-change determined as 2^−ΔΔCt^. Supplementary Table S3 shows the primer sequences used in this study.

### Gene expression omnibus (GEO), gene set enrichment analysis (GSEA) and Venn diagram

For differential gene expression analysis, a search on the Gene Expression Omnibus (GEO) platform was performed (https://www.ncbi.nlm.nih.gov/gds) in order to find studies with microarray analysis comparing non-tumor vs tumor-bearing mice spleens and tumors (B16F10, LLC, CT26 and 4T1). GSE85507 dataset was used^90^. We analyzed GSE85507 dataset using GEOR2 and set adjust p-value < 0.05 as a significant threshold. Comparisons were performed as follows: (1) Spleen non-tumor C57BL6 and Balb/c mice vs Spleen LLC, 4T1 and CT26 tumor-bearing mice, (2) Tumor B16F10 tumor-bearing mice (non-splenomegaly) vs Tumor LLC, 4T1 and CT26 tumor-bearing mice (splenomegaly). Gene set enrichment analysis (GSEA, Broad Institute)^91^ was used to identify biological pathways enriched in the spleen of non-tumor *vs* tumor-bearing mice. Gene sets were obtained from the MSigD “HALLMARK” collection at The Molecular Signature Database; http://www.broadinstitute.org/gsea/msigdb/collections.jsp; http://www.broadinstitute.org/gsea/msigdb. Data generated by GSEA are shown as enrichment score (ES), normalized enrichment score (NES), NOM p-value, and false discovery rate (FDR). The P value was computed using 1000 permutations for each gene set and corrected by the FDR. Enriched biological pathways were defined as FDR < 0.20 and NOM p-value < 0.05. Venn diagram was generated using InteractiVenn^92^.

### Platelet count

Blood was collected into heparinized vials via retroorbital in baseline (before exercise training – non-tumor), post-training (30 days of exercise training – non-tumor) and post-10 days of CT26 tumor cell injection (CT26 tumor-bearing mice). Whole blood samples were subjected to a standard Complete Blood Count (CBC), including Platelet Count (PLT).

### Aspirin treatment

Aspirin, 450 mg (Sigma, A2093-100G), was dissolved in ethanol to make a 100 mg/mL concentrated stock solution. The stock solution was diluted in PBS to make a 5 mg/mL injection solution. Mice were injected intraperitoneally with aspirin at 50 mg/kg (200 uL/mouse) 3 times a week for 4 weeks before the tumor cell injection and for another 12 days after the tumor cell injection.

### Database and statistical analysis

Kaplan–Meier analysis was performed in R2: Genomics Analysis and Visualization Platform (http://r2.amc.nl), or Kaplan–Meier Plotter (http://kmplot.com/)^93^, or Genetic determinants of cancer patient survival (tcga-survival.com). Gene expression analysis across TCGA cancers was generated with RNAseq analysis and visualization platform UALCAN (http://ualcan.path.uab.edu/index.html).

Statistical analyses were performed using GraphPad Prism Software 9.5.1 (San Diego, CA, USA). Quantitative data are presented as mean ± SD and figure legends indicate the number of animals used in each experiment. Two-tailed unpaired Student’s *t* test was used to determine statistical significance when two groups were compared. One-way ANOVA followed by *Post-hoc* Tukey’s test was used to determine statistical significance when multiple groups were compared. Repeated measures Two-way ANOVA followed by *Post-hoc* Tukey’s was used to compare differences in experiments with different time points. Pearson correlation coefficient (r^2^ value) was calculated assuming a linear relationship between variables. Statistical significance was set at p < 0.05.

### Supplementary Information

Supplementary Information.

Supplementary Table S1.

Supplementary Table S2.

## Data Availability

All current study data are available from the corresponding author on request.
