# Supplementary material for: Aerobic exercise training mitigates tumor growth and cancer-induced splenomegaly through modulation of non-platelet platelet factor 4 expression
Source: Sci Rep. 2023 Dec 11;13:21970. doi: 10.1038/s41598-023-47217-2 (PMC10713653; doi:10.1038/s41598-023-47217-2)
Supplement: Supplementary file 1 — Supplementary Information. [file 41598_2023_47217_MOESM1_ESM.docx]

**Supplementary Tables and Figures:**

**Aerobic Exercise Training Mitigates Tumor Growth and Cancer-Induced Splenomegaly Through Modulation of Non-Platelet Platelet Factor 4 Expression**

Gabriel C. Tobias^1,2^*, João L. P. Gomes^1^, Larissa G. Fernandes^1^, Vanessa A. Voltarelli^1,3^, Ney R. de Almeida^1^, Paulo R. Jannig^1,4^, Rodrigo W. A. Souza^1,3^, Carlos E Negrão^1^; Edilamar M. Oliveira^1^, Roger Chammas^5^, Christiano R. R. Alves^1^, Patricia C. Brum^1^*

^1^School of Physical Education and Sport, University of São Paulo, São Paulo, Brazil

^2^Children’s Cancer and Blood Foundation Laboratories, Departments of Pediatrics, and Cell and Developmental Biology, Drukier Institute for Children’s Health, Meyer Cancer Center, Weill Cornell Medicine, New York, NY, USA

^3^Department of Surgery, Beth Israel Deaconess Medical Center, Harvard Medical School, Boston, MA 02115, USA.

^4^Department of Physiology and Pharmacology, Karolinska Institutet, 171 77 Stockholm, Sweden.

^5^Department of Radiology and Oncology, School of Medicine, University of São Paulo, São Paulo, Brazil.

**^*^Correspondence**

Gabriel C. Tobias ([gct4001@med.cornell.edu](mailto:gct4001@med.cornell.edu))

Patricia C. Brum ([pcbrum@usp.br](mailto:pcbrum@usp.br))

Avenida Professor Mello Moraes, 65 - Butantã, 05508-030, Sao Paulo, SP, Brazil.

Tel: +55 11 3091-2149; Fax: +55 11 3813-5921

**Supplementary Table S1:** Differential gene expression in the spleen of tumor-bearing mice.

**Supplementary Table S2:** Differential gene expression in tumor of tumor-bearing mice.

**Supplementary Table S3.** Primer sequences used in this study.

**Supplementary Figure S1.** Wheel running increases the aerobic capacity in C57BL/6 but not in Babl/c mice. **A**. Daily distance in the voluntary exercise training protocol (n=10) and (**B**) distance until exhaustion during a maximal incremental running test before and after the voluntary exercise training protocol in C57BL/6 mice. Untrained (n=10). Trained (n=10). **C**. Daily distance in the voluntary exercise training protocol (n=10) and (**D**) distance until exhaustion during a maximal incremental running test before and after the voluntary exercise training protocol in Balb/c mice. Untrained (n=10). Trained (n=10). Data are presented as mean ± SD. Repeated measures Two-Way ANOVA followed by *post-hoc* Tukey’s was used to compare differences between the variables. *p < 0.05, **p < 0.01, ***p < 0.001*,* ****p < 0.0001. The individual dots represent the data points from each mouse.

**Supplementary Figure S2**. Exercise does not change tumor growth in MMTV-PyMT mice and mouse 4T1 tumors. **A:** Experimental protocol. **B-D:** tumor mass, percentage of mice without tumor, and distance until exhaustion during a maximal incremental running test in 4T1 tumor-bearing mice. **E-M**: lung mass, liver mass, perigonadal WAT mass, kidney mass, right ventricle mass, left ventricle mass, heart heart, body mass, and spleen mass in MMTV-PyMT mice. Data are presented as mean ± SD. Repeated measures Two-Way ANOVA followed by post-hoc Tukey’s, or two-tailed Student’s t-test, or Two-Way ANOVA followed by post-hoc Tukey’s were used to compare differences between the variables. *p < 0.05, **p < 0.01, ***p < 0.001, ****p < 0.0001. The individual dots represent the data points from each mouse.

**Supplementary Figure S3**. Intrinsic aerobic capacity does not affect tumor growth. **A-H:** Correlation analysis between distance until exhaustion during a maximal incremental running test and tumor volume or tumor mass in B16, LLC, CT26, and 4T1-tumor-bearing mice. **I-J:** Correlation analysis between distance until exhaustion during a maximal incremental running test and tumor volume or tumor mass in all tumor models combined. The individual dots represent the data points from each mouse.

**Supplementary Figure S4**. Tissue mass in LLC, B16, CT26, and 4T1-tumor-bearing mice. Data are presented as mean ± SD. n = 6-16. Repeated measures Two-Way ANOVA followed by post-hoc Tukey’s, or two-tailed Student’s t-test, or Two-Way ANOVA followed by post-hoc Tukey’s were used to compare differences between the variables. *p < 0.05, **p < 0.01, ***p < 0.001, ****p < 0.0001. The individual dots represent the data points from each mouse*.*

**Supplementary Figure S5.** Pf4 expression levels in both spleen and tumor are associated only with tumor growth and not splenomegaly. **A-C**: Correlation analysis between Pf4, Ppbp, and F5 mRNA expression in the tumor and spleen mass. **D-F**: Correlation analysis between Pf4, Ppbp, and F5 mRNA expression in the spleen and spleen mass. The individual dots represent the data points from each mouse*.*

**Supplementary Figure S6**. ASP treatment does not affect Pf4 expression in the CT26 tumor. **A**: Pf4 mRNA expression on CT26 cells, CT26 primary tumor cells, and CT26 tumor. **B-F**: CT26 tumor growth, tumor mass, ex vivo tumor volume, spleen mass, and liver mass in tumor-bearing mice treated with aspirin (CT26+ASP). **G**: Pf4 mRNA expression on CT26 tumor in tumor-bearing mice treated with aspirin. **H**: Plasma Pf4 levels measured by ELISA (CT26 TB: CT26 tumor-bearing mice; CT26 CM: CT26 conditioned media). Data are presented as mean ± SD. Repeated measures Two-Way ANOVA followed by post-hoc Tukey’s, or One-ANOVA followed by post-hoc Tukey’s, or two-tailed Student’s t-test were used to compare differences between the variables. *p < 0.05, **p < 0.01, ***p < 0.001, ****p < 0.0001. The individual dots represent the data points from each mouse.

**Supplementary Figure S7**. Pf4 predicts survival in cancer patients. **A**: PF4 gene expression analysis of Genotype-Tissue Expression GTEx (http://www.broadinstitute.org/gtex/) human normal tissue showing that PF4 is highly expressed in blood. **B**: PF4 gene expression analysis of BioGPS (http://biogps.org) mouse normal tissue and cells showing that PF4 is highly expressed in macrophages, bone, and bone marrow. **C-L**: Overall, relapse-free, or disease-free survival based upon median expression of PF4 in neuroblastoma, brain, cervix, Wilms, ovarian, and breast tumors tissue. Kaplan-Meier analysis was performed in R2: Genomics Analysis and Visualization Platform (<http://r2.amc.nl>). **M-Q**: Overall survival based upon median expression of PF4 in sarcoma, pancreatic ductal adenocarcinoma (PDAC), breast cancer, cervical squamous cell carcinoma, and kidney renal cell carcinoma. Hazard ratio (HR) and logrank p values are shown in the upper right of each Kaplan-Meier Plot. Survival analysis was performed using Kaplan-Meier Plotter (kmplot.com).
